# Web Crippling of Pultruded GFRP Profiles: A Review of Experimental, Numerical, and Theoretical Analyses

**DOI:** 10.3390/polym17202746

**Published:** 2025-10-14

**Authors:** Mohamed Ahmed Soumbourou, Ceyhun Aksoylu, Emrah Madenci, Yasin Onuralp Özkılıç

**Affiliations:** 1 Department of Civil Engineering, Necmettin Erbakan University, 42090 Konya, Türkiye; medahmedsoumbourou@gmail.com; 2 Department of Civil Engineering, Konya Technical University, 42250 Konya, Türkiye; caksoyluktun@gmail.com; 3 Department of Technical Sciences, Western Caspian University, Baku 1001, Azerbaijan; 4 Department of Unique Buildings and Constructions Engineering, Don State Technical University, Gagarin Sq. 1, 344003 Rostov-on-Don, Russia

**Keywords:** pultruded GFRP, web crippling, finite element modeling, failures

## Abstract

Glass fiber reinforced polymer (GFRP) composite profiles produced by pultrusion method are widely used as an alternative to traditional building materials due to their lightness and corrosion resistance. However, these materials are susceptible to crushing type fractures known as “web crippling” especially under local loading due to their anisotropic structure and limited mechanical strength. Understanding web-crippling behavior is crucial for the safe and efficient structural application of pultruded GFRP profiles. This study report narrated the review of experimental, numerical, and analytical investigations of web-crippling behavior of pultruded GFRP profiles. Highlights of the major findings include profile geometry and detailing of the flange–web joint, loading types (end-two-flange (ETF), interior-two-flange (ITF), end bearing with ground (EG), interior bearing with ground (IG)), bearing plate dimensions, presence of web openings, and elevated temperatures. It also considers the limitations of current standards, along with new modeling techniques that incorporate finite element analysis as well as artificial intelligence. Damage types such as web–flange joint fractures, crushing, and buckling were comparatively analyzed; design approaches based on finite element modeling and artificial intelligence-supported prediction models were also included. These insights provide guidance for optimizing profile design and improving predictive models for structural engineering applications. Gaps in current design standards and modeling approaches are highlighted to guide future research.

## 1. Introduction

Glass fiber reinforced polymer (GFRP) composites are increasingly employed in the construction, marine, transportation, and energy sectors due to their favorable characteristics, including low weight, corrosion resistance, high specific strength, and electromagnetic shielding capability [1,2,3]. Furthermore, GFRP composites are being utilized more and more often in maritime settings for a wide range of applications, including drilling platforms and caissons in seaports, in order to solve the problem of corrosion that is associated with conventional steel reinforcement [4,5,6]. Additionally, it is utilized for various construction industries due to their prementioned superior properties [7,8,9].

Notably, pultrusion, a widely adopted manufacturing method for these composites, offers continuous profile geometry, high reproducibility in production, and cost-effectiveness, among other benefits [10,11]. Pultrusion is carried out by pulling glass, carbon, or aramid fibers through a guide and by precisely distributing them according to the cross-section of the profile [12]. As a consequence of the use of carbon, boron, glass, and aramid fibers in conjunction with ceramic and metal matrices, a large number of composite material applications that are still in the process of being developed have been discovered in recent years [13]. In the literature, there are a number of research that have been conducted on experimental studies of pultruded FRP composite constructions [14,15,16].

Among the various methods used in the production of fiber-reinforced polymer composites, pultrusion is a prominent technique that enables the continuous, automated, and efficient production of constant-section profiles. Compared to methods such as hand layup and resin transfer molding, pultrusion is less labor intensive, offers greater dimensional consistency, and offers production repeatability. Furthermore, while filament winding is generally limited to cylindrical and closed geometries, pultrusion allows for the production of a wide variety of open and closed profiles with I, C, Z, and box cross-sections. Thanks to the continuous production process, pultrusion-produced components are generally more suitable and economical for long, flat, structural elements such as beams, gratings, and staircases. Furthermore, by integrating fiber bundles, surface covers, and multi-directional reinforcements, profiles can be optimized for specific loading conditions [17,18,19].

**Figure 1:**
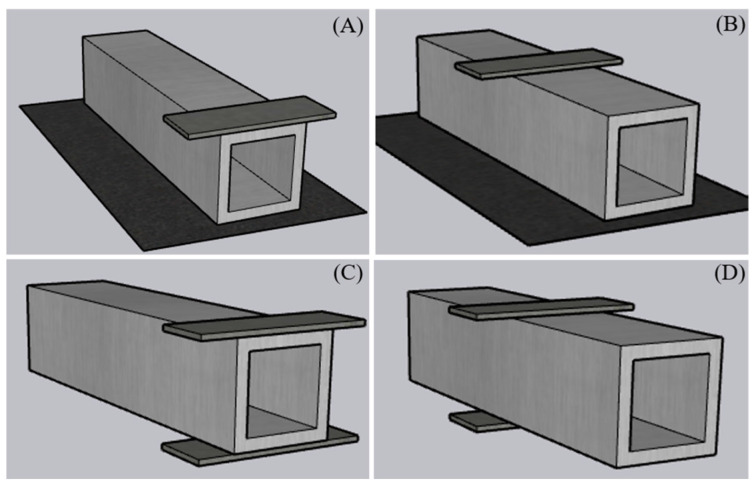
Representation of loading conditions. (**A**) End bearing with solid ground (EG), (**B**) interior bearing with solid ground (IG), (**C**) end two flange (ETF) and (**D**) interior-two-flange (ITF).

Pultruded GFRP profiles can be fabricated in a variety of cross-sectional shapes including I, C, Z, and box sections and are particularly used in short-span structural members [20,21,22,23,24]. However, the anisotropic nature and limited shear stiffness of these profiles lead to mechanical vulnerabilities under certain loading scenarios. In this regard, one of the most critical failure modes, web crippling, manifests as localized crushing, buckling, or cracking in the web region under concentrated bearing or local compression loads. Web crippling typically initiates at the flange–web junction and, depending on the loading configuration, may develop into various failure modes [25,26,27,28,29,30,31,32,33]. The literature highlights four primary loading configurations: end-two-flange (ETF), interior-two-flange (ITF), end bearing with ground (EG), and interior bearing with ground (IG). These configurations directly influence the failure mode and load-carrying capacity of the web (Figure 1).

Web crippling is influenced not only by the loading configuration but also by profile geometry, web thickness, bearing plate dimensions, opening locations, and temperature effects [34,35]. Studies evaluating these multi-parameters indicate that pultruded GFRP profiles exhibit markedly different behavior compared to isotropic materials such as steel or aluminum [20]. Consequently, the direct application of existing design standards developed for steel structures to pultruded GFRP profiles introduces significant uncertainties. Experimental investigations of web crippling have predominantly focused on short-span I- and C-section profiles [36]. These studies are particularly important for evaluating the effects of the web–flange joint, fiber orientation, carrier plate width, and opening edge detailing. Several investigations have demonstrated that the fiber reinforcement of opening edges can substantially increase the crippling load [37].

Numerical studies employing finite element modeling (FEM) have significantly advanced the identification of distinct damage modes and the analysis of stress distributions of composite materials [38,39,40,41,42]. Recently, data-driven approaches such as artificial intelligence and machine learning have been increasingly employed to predict the behavior of composite materials [43,44,45,46,47]. Although these approaches can achieve higher accuracy than traditional analyses, they remain dependent on large volumes of experimental data.

Moreover, environmental factors such as humidity and saltwater exposure substantially influence the crippling behavior of GFRP [6,34]. Considering these effects during the design phase is critical, particularly in aggressive environments such as marine structures or chemical plants. Furthermore, modifications like creating openings in C- or I-section profiles (e.g., for cable routing) induce stress concentrations in the web region, thereby reducing the crippling load.

This study reviews the existing literature on the web-crippling behavior of pultruded GFRP profiles, aiming to provide practical guidance for both engineers and researchers. It comprehensively details how variables such as loading configurations, profile geometry, clearance and bearing conditions are addressed through experimental, numerical, and theoretical approaches; the shortcomings of current practices are critically examined, and directions for future research are proposed.

## 2. Experimental Evidence on Web-Crippling Behavior of Pultruded GFRP Profiles

### 2.1. Effect of Profile Geometry on Web-Crippling Behavior of Pultruded GFRP Profiles

**Figure 2:**
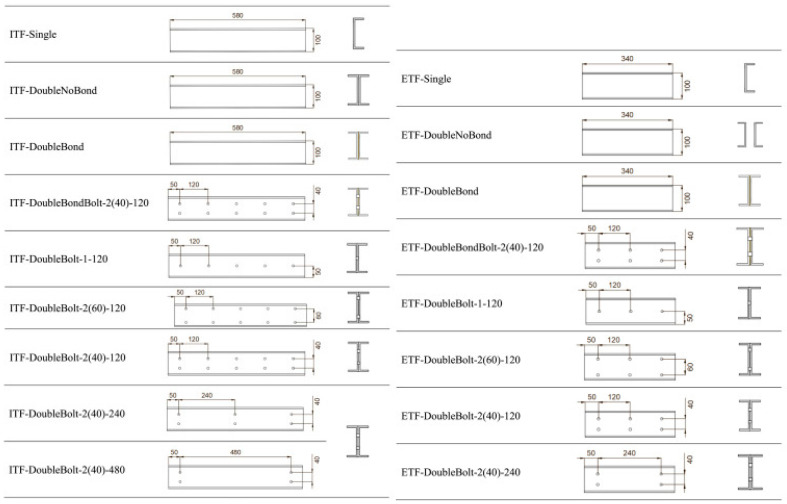
The specimens of the experimental study conducted by Wu et al. [15].

Pultruded GFRP sections can be fabricated in a wide variety of shapes, analogous to those used in steel structures. Manufacturers typically fabricate pultruded GFRP profiles in open cross-sections such as I-, H-, L-, and U-sections and in closed cross-sections, including square, rectangular, and circular shapes [48]. In studies on web-crippling behavior, many researchers have focused on I-shaped profiles, rectangular hollow sections (RHS), and U-shaped channels. Among these, the U-shaped channel represents a relatively simpler structural form compared to I-shaped and RHS profiles. U-shaped channels exhibit single-axis symmetry, whereas I-shaped and RHS profiles generally possess double-axis symmetry. In certain cases, double-axis symmetry can also be achieved by pairing two U-shaped channels. When transverse loads are applied, particularly through the flanges, these profiles may experience web crippling due to stress concentration in the web region. A typical study by Wu et al. [15] compared the web-crippling behavior of single U-channels with various I-section configurations, including adhesively bonded and mechanically bolted sections. Five types of specimens were tested: a single U-channel; two U-channels forming an I-section without connection; an adhesively bonded I-section; a mechanically bolted I-section; and an I-section with both adhesive bonding and mechanical bolting. For the mechanically bolted sections, the positions and spacing of the bolts were varied (Figure 2). All specimens were tested under ETF and ITF loading conditions. The ultimate load was highest for the adhesively bonded and mechanically bolted I-sections under ITF, and for the mechanically bolted I-section under ETF. Observed failure modes included web–flange junction (WJF) failure at the inner corners for a single U-channel and unconnected built-up sections under both ETF and ITF, as well as web cracking around the bolt holes in bolted sections. Crushing behavior in pultruded GFRP profiles is directly related not only to the external geometry but also to the damage mechanisms associated with the internal structure. Stress concentrations occurring at the web–flange junction often cause this area to be the first to suffer damage. Due to the brittle nature of the resin, damage types such as matrix cracking, followed by fiber fracture and delamination are observed in these areas. Especially in thin-walled and asymmetrical sections, high shear stresses under loading can lead to 45°-angle cracks. These cracks are initially localized, but as the load increases, crack propagation spreads along the web, causing sudden crushing or buckling of the profile. The common features of the two built-up I-sections exhibiting high ultimate load capacity are the mechanical bolt connections and the number of vertical bolts. The study also highlights the influence of web openings in PGFRP beams, which help redistribute applied forces to the surrounding material. While perforations in the web offer architectural advantages, as noted in the introduction, they generally reduce the shear capacity of the beam, whereas the flanges primarily provide flexural resistance.

**Figure 3:**
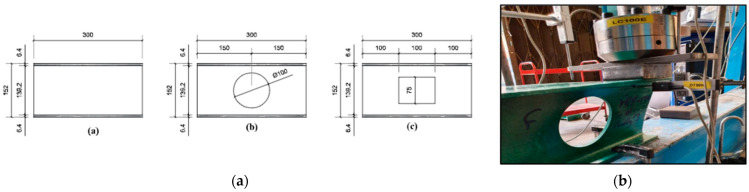
Pultruded GFRP I-section beams: (**a**) I—pultruded GFRP sections with different shapes of holes; (**b**) ETF loading conditions for I-section with circular opening (conducted by Gand et al. [49]).

**Figure 4:**
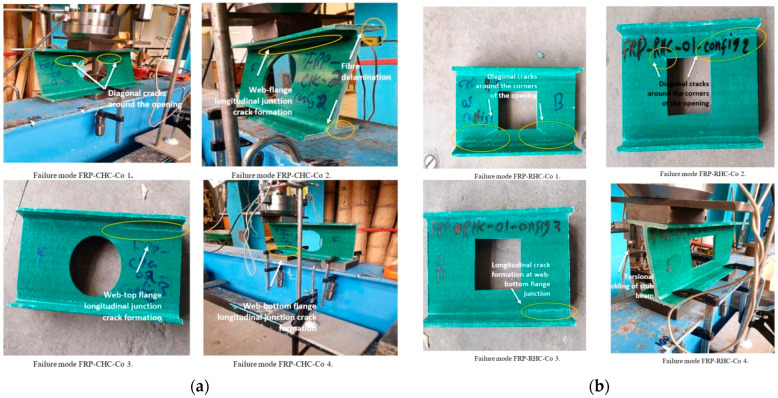
Failure modes of pultruded GFRP I-section beams: (**a**) failure modes of circular openings; (**b**) failure modes of rectangular openings (conducted by Gand et al. [49]).

Gand et al. [49] conducted an experimental study investigating the performance of pultruded GFRP I-section beams with square and circular holes on web crushing behavior (Figure 3a). The beams were subjected to ETF, ITF, EG, and IG loading conditions (Figure 3b). The presence of holes significantly affected the beam strength, with square holes reducing strength by 25% and circular holes by 20%. Failure modes for beams with circular and rectangular openings are shown in Figure 4a and Figure 4b, respectively. While the primary failure mode for circular openings was web–flange junction (WJF) failure, the rectangular openings exhibited WJF accompanied by diagonal cracks at the corners.

**Figure 5:**
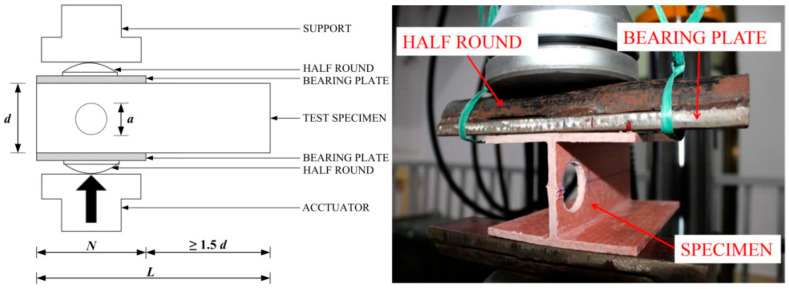
Test setup of ETF loading condition for I-section with circular opening conducted by Haloi et al. [50].

**Figure 6:**
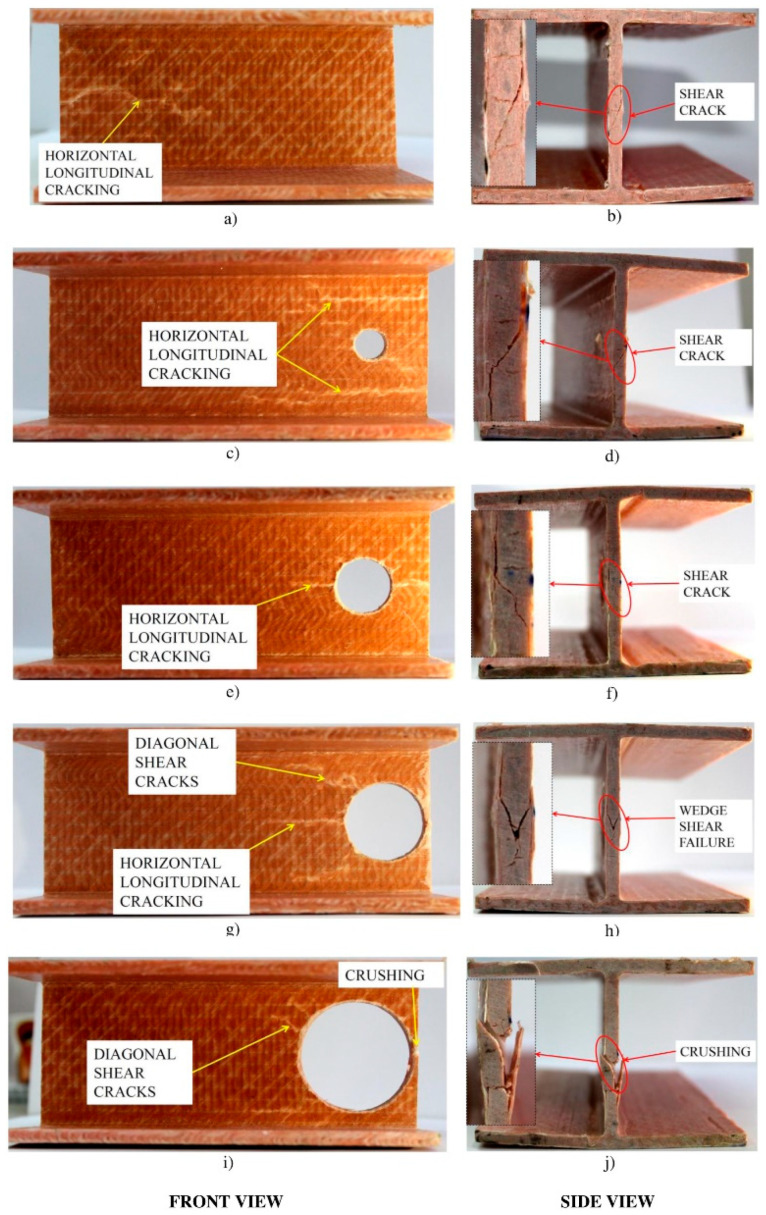
Observed failure modes for I-section with circular opening conducted by Haloi et al. [50].

Haloi et al. [50] evaluated the web-crippling performance of pultruded GFRP wide-flange section profiles with and without perforations. In this study, perforation sizes were varied from 0.2 h to 0.8 h to better understand their effects, and the specimens were subjected to ETF loading (Figure 5). Increasing perforation diameters resulted in a reduction in ultimate load ranging from 26% to 78.3%. It was also reported that the failure modes depend on the size of the web openings (Figure 6).

**Figure 7:**
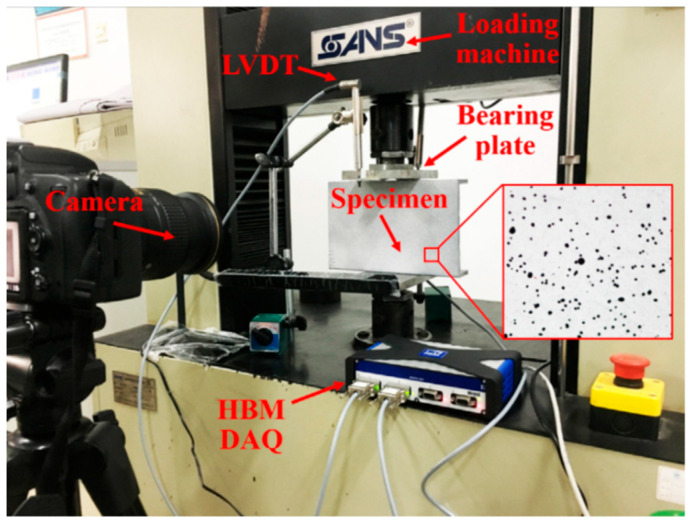
Test setup of Wu et al. [20].

**Figure 8:**
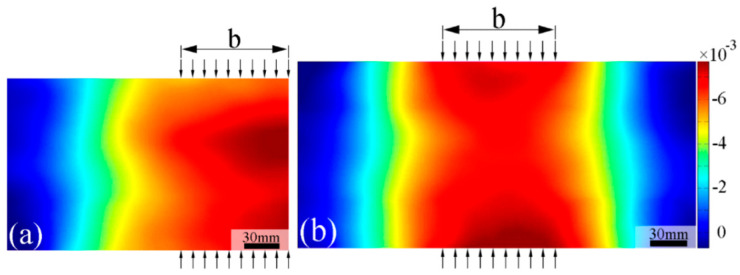
DIC of Wu et al. [20].

**Figure 9:**
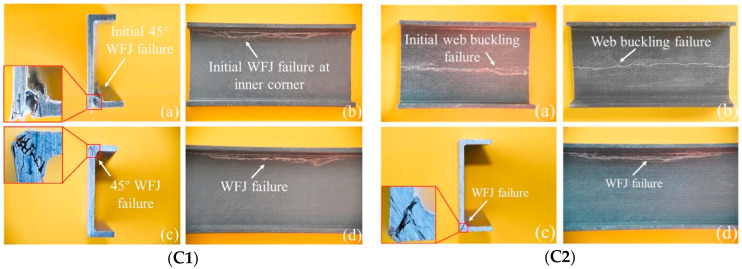

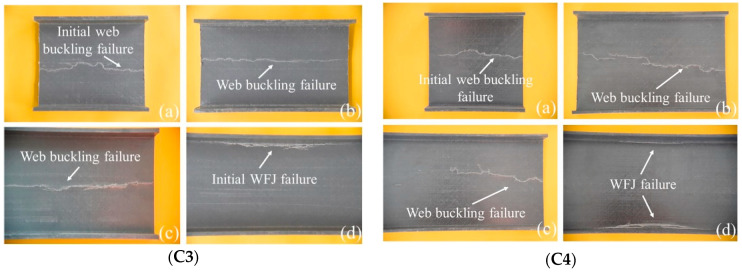
Observed failure modes of channel sections conducted by Wu et al. [20].

According to numerous studies, the geometric shape of pultruded GFRP profiles can significantly influence their web-crippling behavior. Wu et al. [20] investigated four channel sections of pultruded GFRP to analyze the effects of specimen length, sectional dimensions, and loading conditions on web-crippling behavior. The specimens were subjected to ITF and ETF loading conditions, as illustrated in Figure 7, and digital image correlation (DIC) measurements were also performed (Figure 8). Increasing the web slenderness ratio and specimen length, or changing the loading from ITF to ETF, significantly affected web-crippling behavior. Initial damage, observed as cracking at 45° web–flange junctions, occurred on C1 specimens due to loading conditions and length; on C2 specimens due to length, particularly in long samples; and on C3 and C4 specimens under ITF loading. Crack propagation along the web length was faster in short specimens compared to the long C2 specimens. For other specimens, initial cracking occurred at the mid-height of the web, and some, such as the short C2 specimens, even exhibited local buckling (Figure 9). 

### 2.2. Effect of Bearing and Loading on Web Crippling of Pultruded GFRP

**Figure 10:**
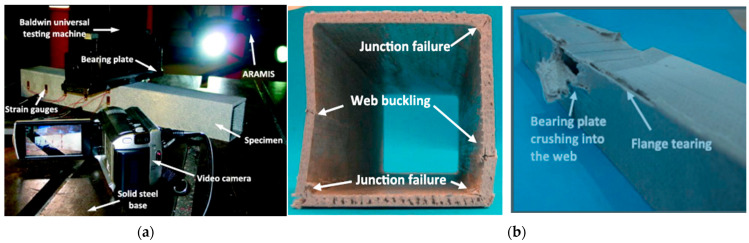
Application of bearing plate and its effect on the specimen: (**a**) test setup used by Wu and Bai [37]; (**b**) damages on pultruded GFRP hollow sections [37].

Bearings are essential for the stability of pultruded GFRP profiles, as the sections cannot remain stable without them. The position and application of the bearings (ETF, ITF, EG, and IG loading type) are critical for web-crippling tests, as they directly influence the observed failure modes. However, most studies indicate that the geometric shape of pultruded GFRP sections significantly influences web damage. Specimen length is one of the most frequently cited parameters in the literature [51,52]. An initial investigation by Wu and Bai [37] examined the effects of bearing length on the web-crippling behavior of pultruded GFRP profiles. Four groups of square hollow sections with different dimensions were subjected to ETF, ITF, EG, and IG loading, with concentrated loads applied through 50 mm long steel bearing plates (Figure 10a). All specimens exhibited initial web–flange joint failure under these loading conditions (Figure 10b). The study demonstrated that web-crippling capacity is related to interlaminar shear stress, the shear zone beneath the bearing plates, and the distribution of shear stresses.

**Figure 11:**
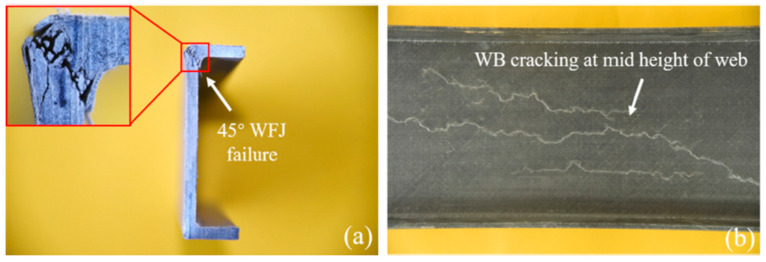
Observed failure modes of channel sections conducted by Wu et al. [31].

Wu et al. [31] investigated the effects of bearing length on the web-crippling behavior of pultruded GFRP channel sections. Four channel sections with varying bearing lengths were tested under ETF and ITF loading conditions using the setup shown in Figure 7. The results indicated that the web-crippling capacity is strongly influenced by bearing length. Under ETF conditions, increasing bearing length led to capacity increases of up to 496.9%, 498.0%, 105.7%, and 45.3% for sections C1 to C4, respectively, while under ITF conditions, the corresponding increases were 355.7%, 361.4%, 49.9%, and 62.4%. Increasing the bearing length also led to reductions in web-crippling capacity under all loading conditions, particularly under ETF. Failure modes ranged from web–flange junction (WFJ) to web buckling (WB) for C3 and C4 under ITF with increasing bearing length, whereas only WFJ failure was observed for C1 and C2 under both ETF and ITF conditions (Figure 11).

**Figure 12:**
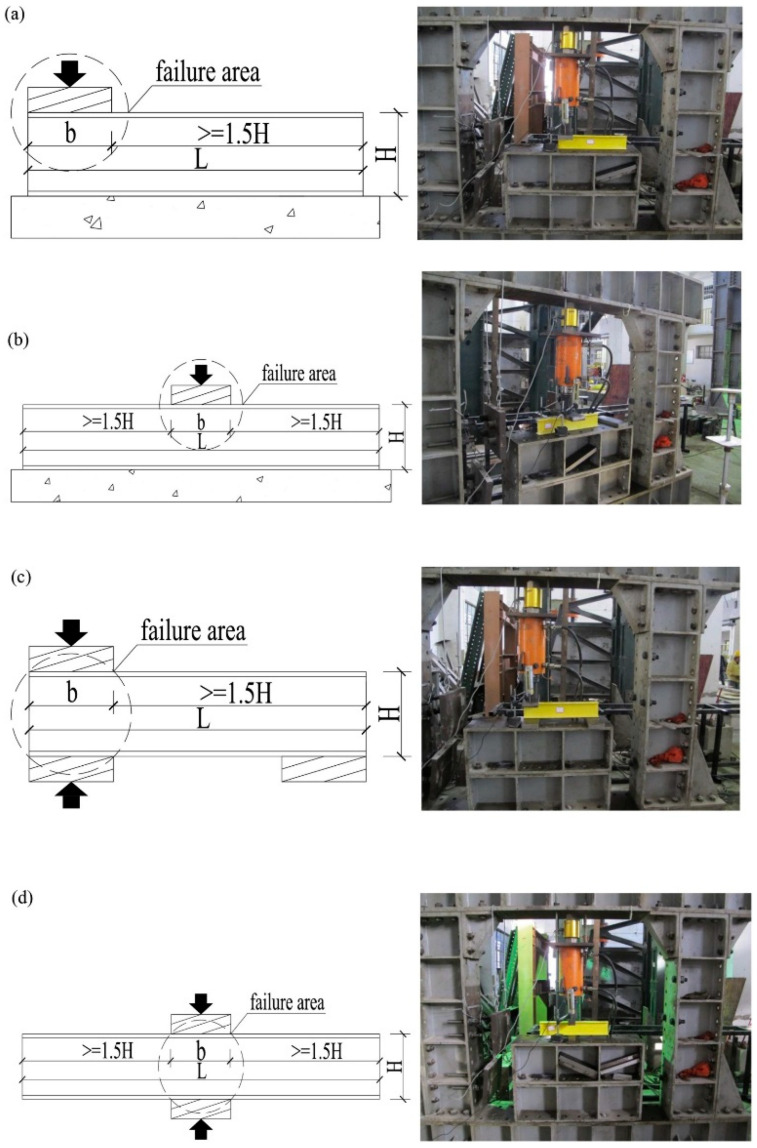
Test setup and loading conditions of Chen and Wang [53]: (**a**) end bearing with solid ground; (**b**) interior bearing with solid ground; (**c**) end-two-flange; (**d**) interior-two-flange.

**Figure 13:**
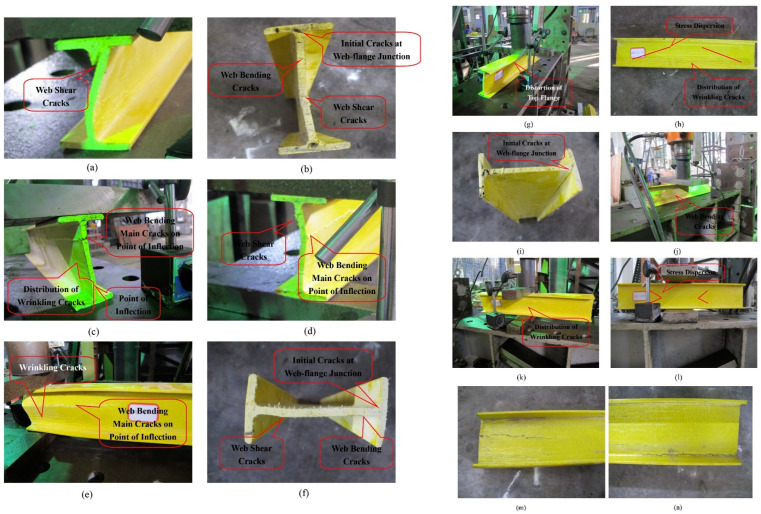
Observed failure modes of channel sections conducted by Chen and Wang et al. [53].

In another study, Chen and Wang [53] investigated the web-crippling behavior of pultruded GFRP I-sections, focusing on the effects of loading conditions and bearing length on both web crippling and ductility. Twelve pultruded GFRP sections with varying bearing lengths were tested under four loading configurations: ETF, ITF, EG, and IG (Figure 12). Initial web–flange junction (WFJ) failure was observed in all specimens, followed by other failure modes such as bending cracks and shear cracks under EG and ETF, and top flange distortion under IG (Figure 13). The study showed that specimens were generally more resistant under interior loading conditions, exhibiting higher ultimate loads compared with end loading. Furthermore, large variations in bearing plate length generally led to reductions in ultimate load, except under IG conditions. The observed failure modes for the tested specimens are summarized in Figure 13.

Chen and Wang [54] tested pultruded GFRP rectangular hollow sections under four distinct loading conditions using the same experimental setup shown in Figure 12. Three bearing lengths (50 mm, 100 mm, and 150 mm) were employed to apply the concentrated loads. The authors proposed a set of unified formulas based on Eurocode 3, which accurately predicted the experimental results.

**Figure 14:**
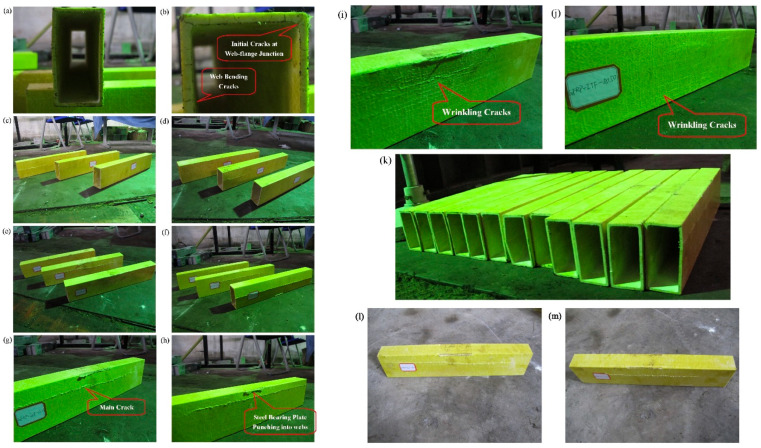
Observed failure modes of hollow box sections conducted by Chen and Wang et al. [54].

The observed failure modes are illustrated in Figure 14. Two main failure patterns were identified depending on the initial crack and loading type: for webs exhibiting initial outward bending, a primary longitudinal crack forms at the center of the web accompanied by wrinkling cracks near the web–flange junction; for webs with inward bending, only longitudinal wrinkling cracks develop, covering nearly the entire web without a primary crack. Specimens with inward bending showed higher web-crippling capacity, likely due to the stiffer boundary conditions of the web. Additionally, the bearing plates caused local cracks during interior loading, as shown in Figure 14.

**Figure 15:**
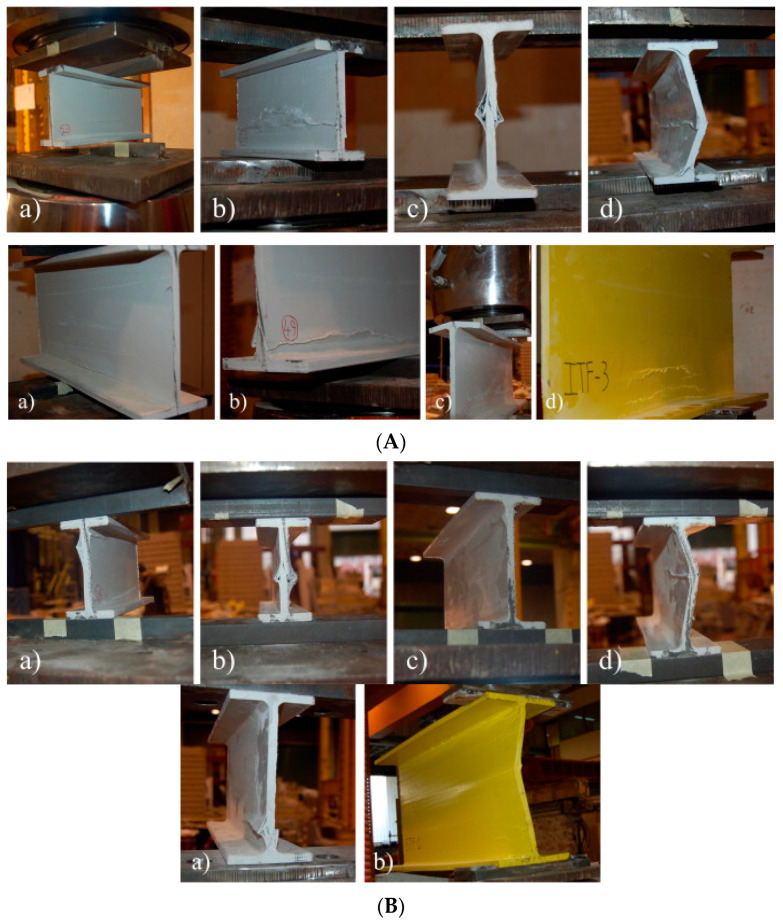
Observed failure modes of I-sections conducted by Fernandes et al. [10]: (**A**) ITF failure modes; (**B**) ETF failure modes.

Fernandes et al. [10] presented both experimental and numerical studies on the web-crippling behavior of pultruded GFRP profiles (Figure 15). Four different profiles with heights ranging from 100 mm to 400 mm were tested, and three bearing lengths (15 mm, 50 mm, and 100 mm) were employed. The experimental results showed that increasing the bearing length from 15 mm to 100 mm led to a rise in web-crippling deformation capacity by 139% and 230% for ITF and ETF loading conditions, respectively, while stiffness increased by 129% and 224%, respectively. Two main types of failure were observed under both loading conditions: web crippling and web buckling (Figure 15A,B). Failures were characterized by longitudinal cracking and material wrinkling, occurring either at the web–flange junctions or at mid-web height. For smaller sections such as I100 and I120, crack locations varied with bearing length: short bearings caused failures at the web–flange junction (Figure 15Aa), while long bearings induced failures at mid-web height (Figure 15Ab). In contrast, for larger sections, failure locations were generally independent of bearing length. Furthermore, the study highlighted that large-section profiles, particularly those with substantial web depth, were more prone to initial failures.

Similarly, Zhang and Chen [30] investigated the effects of loading positions, support conditions, and bearing length on the web-crippling behavior of twelve pultruded GFRP channel sections. Specimens were tested under ETF, ITF, EG, and IG loading configurations with varying bearing lengths. The results showed that when the load was positioned near the center, particularly under IG loading, the load distribution was more uniform, resulting in higher ultimate strength. However, variations in support length did not produce consistent trends; in particular, ultimate strength did not necessarily increase with longer supports.

**Figure 16:**
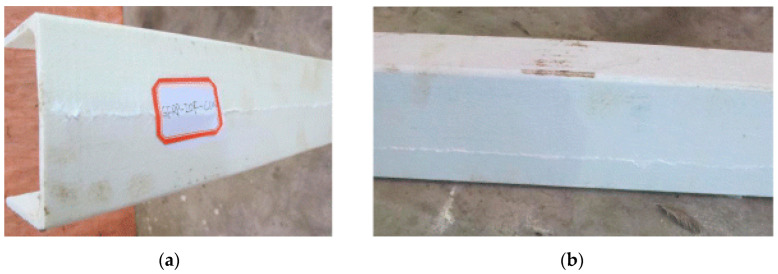
Observed failure modes of channel sections conducted by Zhang and Chen et al. [30]: (**a**) IG-100; (**b**) ITF-50.

Under different loading conditions, the specimens primarily failed due to a main bending crack. Before the cracks propagated along the entire length of the samples, flange separation occurred at the bearing plate and at the ground. Ultimately, the web fractured longitudinally at the center. Specimens were more severely affected when seated directly on the ground (Figure 16).

**Figure 17:**
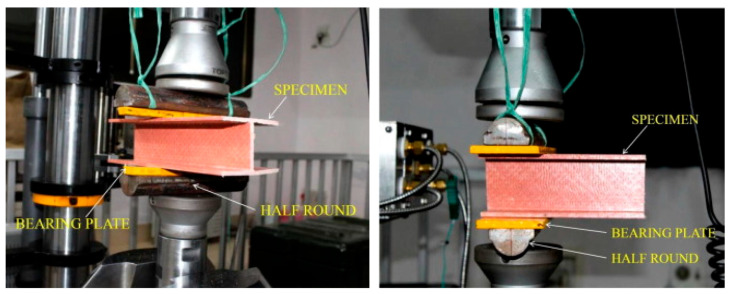
Test setup of Haloi et al. [36].

**Figure 18:**
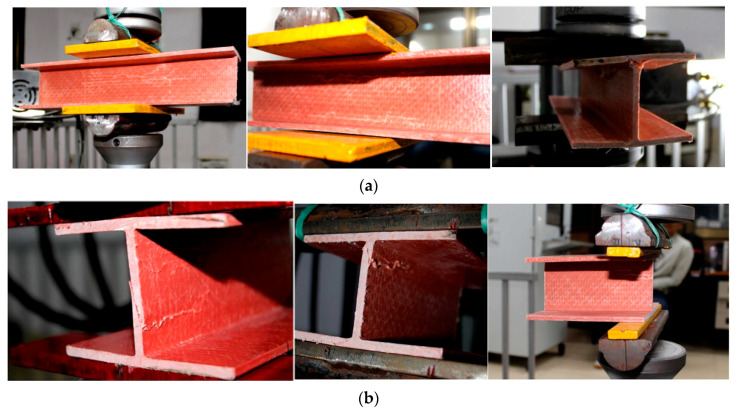
Observed failure modes of I-sections conducted by Haloi et al. [36]: (**a**) ITF samples; (**b**) ETF samples.

Haloi et al. [36] investigated the combined effects of loading condition, bearing length, and plate thickness on the web-crippling behavior of pultruded GFRP wide-flange (WF) sections. A total of 26 WF sections with varying bearing lengths and bearing plate thicknesses of 8 and 10 mm were tested under ITF conditions, while an additional 27 specimens were tested under ETF loading configurations (Figure 17). Under ITF loading, early cracks appeared at approximately 45° at the WFJ, accompanied by longitudinal cracks or material wrinkling at the same junction (Figure 18a). Under ETF loading, the specimens exhibited a sharply sloping “shear wedge” in their cross-sections, extending from the end toward the interior (Figure 18b). The study showed that bearing length has a significant effect on the web-crippling strength and behavior of WF sections, whereas the influence of bearing plate thickness on web-crippling strength is relatively minor.

**Figure 19:**
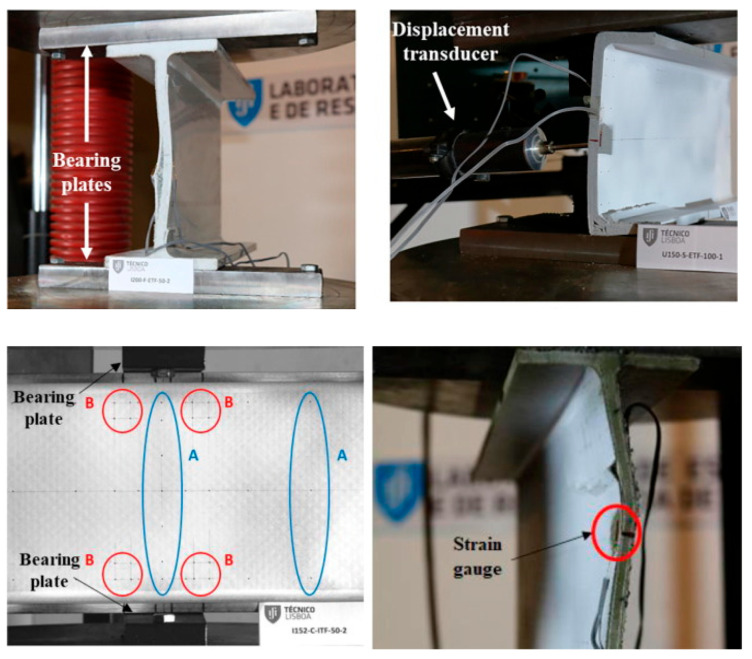
Test setup of Almeida-Fernandes et al. [28].

**Figure 20:**
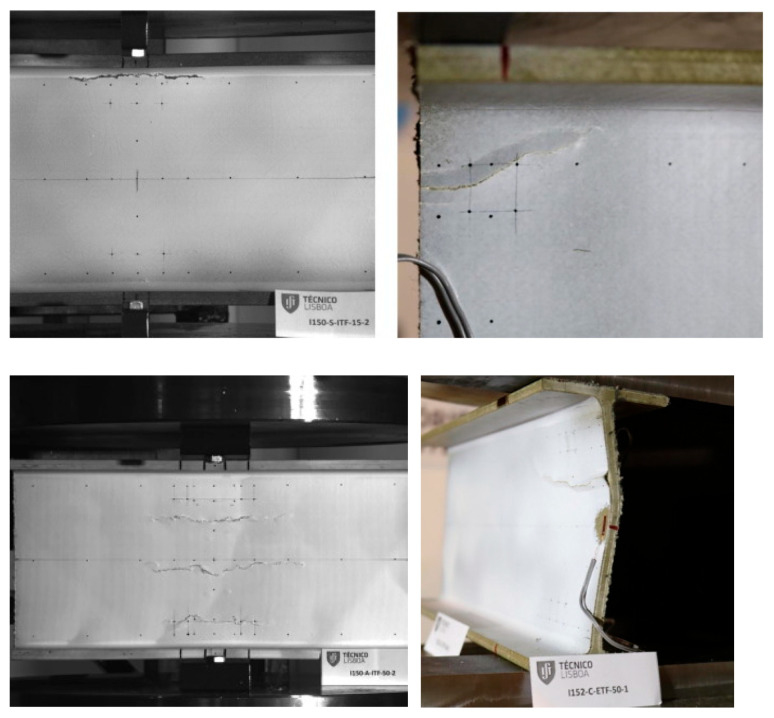
Observed failure modes of I-sections conducted by Almeida-Fernandes et al. [28].

Almeida-Fernandes et al. [28] examined the influence of fiber orientation and bearing length on the web-crippling behavior of pultruded GFRP profiles. Tests were conducted on GFRP sections with varying fiber layups and bearing lengths under ETF and ITF loading scenarios (Figure 19). Despite considerable variations in fiber layups, no significant effects on stiffness or ultimate load were observed. In contrast, bearing length had a pronounced impact on failure modes, stiffness, and ultimate load across all I-section designs. Interestingly, all I-section profiles exhibited similar transverse compressive strain distributions under identical loading conditions, suggesting that fiber layup variations had negligible influence on the effective bearing length. The predominant damage modes observed were web crushing and web buckling (Figure 20).

The width and thickness of the bearing plates are additional critical parameters influencing web crippling. Borowicz and Bank [55] conducted a detailed study on pultruded GFRP I-sections subjected to concentrated loads, examining eight specimens with varying bearing widths and thicknesses and twelve specimens without bearing plates. A total of 20 specimens were evaluated under three-point bending. Most specimens exhibited wedge-like shear failures at the top WFJ, with cracks propagating along the beam length. Bearing plates had a significant effect on web crippling; increasing plate thickness enhanced the load-bearing capacity by approximately 17%, whereas plate width had a negligible impact. In a previous study, Borowicz [56] performed similar experiments with different sample quantities, including 24 pultruded GFRP beams without bearing plates and 13 beams with bearing plates of varying widths and thicknesses. In all cases, failure was initiated at the upper flange junction near the applied load due to interlaminar shear, forming a ‘V’-shaped wedge into the web. This was accompanied by localized buckling at the top of the web in the immediate load zone and longitudinal cracking along the flange–upper web junction. The same failure modes were observed with and without bearing plates, though they were more pronounced in specimens with bearing plates. Overall, the studies confirmed that bearing plate thickness plays a major role in enhancing web crushing resistance, while plate width has a lesser effect.

The loading configurations used in the studies (ETF, ITF, EG, IG) represent both the conditions encountered in structural applications and the loading scenarios. These configurations simulate various situations, such as overlapping the support point, a point load at the center of the span, and a force applied to the flange edge, thus modeling the loading conditions encountered in real-world engineering applications. The location of the load, such as at the end or mid-span, significantly alters the boundary conditions and stress distribution. The load type (point, distributed, or eccentric) influences the regions where stress concentrations occur. Furthermore, the load magnitude and bearing plate width directly influence the occurrence of failure modes, particularly matrix cracking and fiber separation at the interface. Smaller plate widths increase the risk of delamination by increasing local stress, while larger plates retard deformation by spreading the load over a wider area. Accurate modeling of these effects is critical for the safe design of GFRP members.

### 2.3. Effect of Thermal Variations on Web Crippling of Pultruded GFRP

Thermal variations are an important consideration in any construction material. While reinforced concrete structures generally exhibit higher resistance compared to wood or steel, pultruded GFRP profiles are characterized by low thermal resistance. The fiber and matrix components play a critical role in the thermal response: although GFRP can retain considerable strength at elevated temperatures, most polymer matrices become brittle due to glass transition and degradation processes. The thermophysical and mechanical properties of polymer matrices are significantly affected by temperature [57,58].

The thermoset resins that are in GFRP profiles made by pultrusion reach a certain temperature Tg that begins to cause a loss of hardness [59]. This temperature usually varies from 60 °C to 120 °C and severely compromises the structural and load-bearing properties of the resin. Above Tg, the resin matrix becomes plastic, reducing shear rigidity, weakening stress transfers, and speeding crack growth. Experimental investigations have indicated that, at these temperatures, profiles attain their loss in load capacity suddenly, and crushing damages become more localized and quickly. Because of the increase in temperature weakening the bonds at the interface between the glass fiber and the resin matrix, this leads to micro-level residual stresses generated in these regions owing to the difference in the coefficients of thermal expansion of the fibers and the matrix. These stresses eventually cause debonding and delamination—even under depletion conditions, microcracks originating from these weakened interfaces combine because of heating to form macro-level damages. In this way, profiles can be locally deformed in the web area much more easily, whereby a major reduction in crush strength occurs.

**Figure 21:**
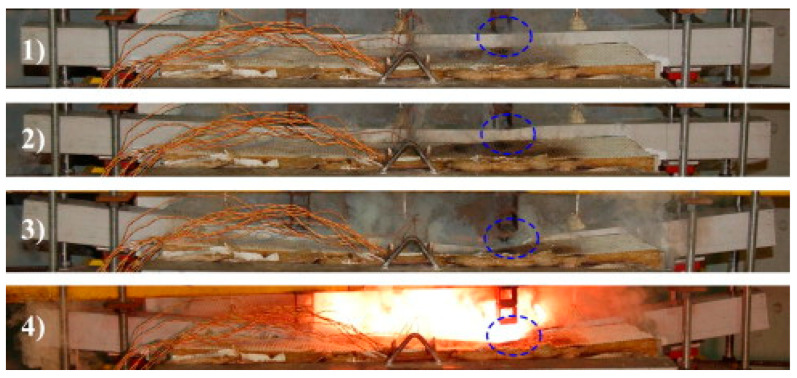
Failure modes of Correia et al. [63].

Jafari et al. [60] demonstrated that thermal cycling significantly affected resin strength while having a minor effect on fiber strength. Their study included pultruded GFRP I- and U-sections tested in three-point bending along weak and strong axes, as well as box sections and laminates tested under compression and tension, all subjected to thermal cycles between −20 °C and 20 °C. Correia et al. [61] also investigated the mechanical behavior of pultruded GFRP, including tensile, shear, and compressive properties, over a temperature range of 20 °C to 250 °C. These studies consistently showed that the mechanical performance of pultruded GFRP degrades at elevated temperatures, particularly under shear and compressive stresses, primarily due to the resin’s glass transition. Thermomechanical studies aimed at predicting the thermal and mechanical responses of pultruded GFRP profiles under fire have attracted significant research interest, with numerous experimental investigations conducted [62,63,64]. A study conducted by Ludwig et al. [64] examined the strength of pultruded GFRP I-sections exposed to high temperatures, considering the effects of time and applied load. At temperatures of 50 °C, specimen strength decreased, and localized buckling occurred in the compressed flange, propagating subsequently into the web. Correia et al. [63] conducted a similar study on fiberglass box beams, where the pultruded GFRP surface was exposed to fire. After 65 min, the specimens failed due to fracture at the top flange accompanied (Figure 21).

**Figure 22:**
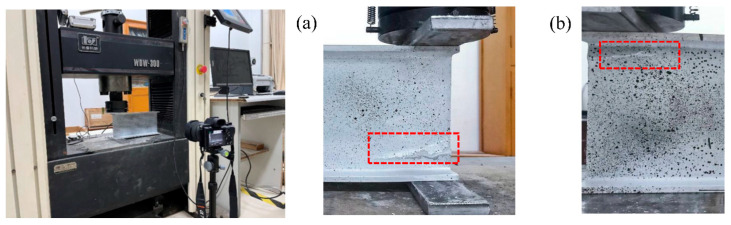
Experimental test setup and failure mode of Zhang et al. [11].

**Figure 23:**
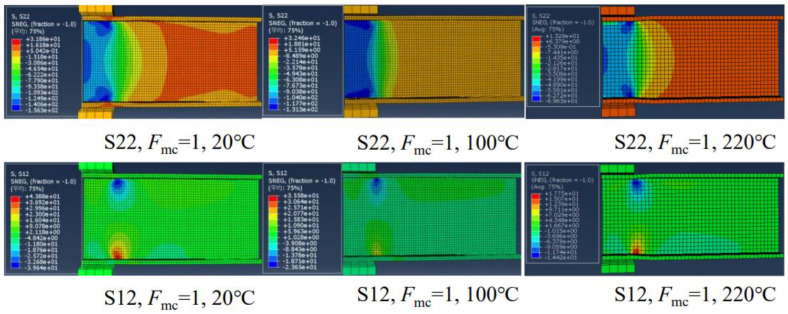

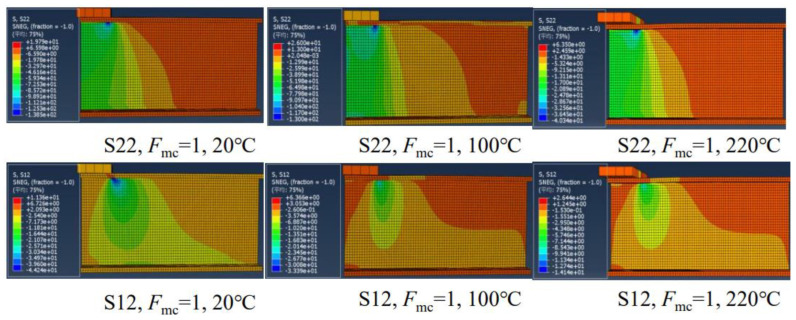
Compressive stress and in-plane shear stress under elevated temperatures reported by Zhang et al. [11].

Zhang et al. [11] numerically investigated the effect of elevated temperatures on the web-crippling behavior of pultruded GFRP I-sections. Specimens were subjected to ETF and EG loading conditions at room temperature (Figure 22). Initial damage was observed at the web–flange junction near the corners of the bearing plates, leading to a reduction in shear strength web-crippling failure, characterized by longitudinal cracks at the WFJ, was the most frequently observed failure mode under all loading conditions. The study also indicated that web-crippling deformations were less pronounced in specimens subjected to EG loading compared to ETF prior to ultimate failure. Later, a numerical analysis was conducted in order to investigate the effects of elevated temperature (Figure 23). The results showed that ultimate loads and stiffness decreased markedly with increasing temperature across all loading conditions.

The experimental studies discussed above are summarized in Table 1.

## 3. Numerical and Analytical Modelling Techniques

**Figure 24:**
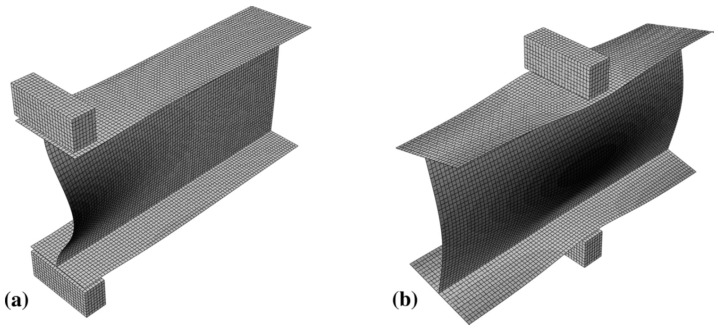
FEA illustration of buckled shape under: (**a**) ETF loading condition; (**b**) ITF loading condition. Fernandes et al. [70].

Following experimental investigations, it is generally recommended to complement them with numerical and analytical analyses to better understand parameters such as stress distributions, failure modes, and load–displacement behavior in pultruded GFRP materials. Numerical methods involve solving problems on a computer, with detailed small-scale modeling of the structure, while analytical methods rely on solving problems using mathematical equations. These approaches provide significant advantages, including the capability to address complex geometries, material nonlinearities, and intricate boundary conditions that are difficult or impossible to handle analytically [68,69,70,71,72]. In most studies, finite element analysis (FEA) models in ABAQUS have been employed to simulate the web-crippling behavior of GFRP sections. Fernandes et al. [70] utilized models incorporating linear buckling analysis, nonlinear analysis, and stress field evaluation. Their results indicate that buckling modes are strongly influenced by the loading configuration (ETF or ITF), and that sections become increasingly sensitive to buckling as the bearing length increases (Figure 24). The Tsai–Hill criterion, although conservative, was used to identify failure zones. Finally, an analytical formula was proposed to predict the failure load of GFRP sections.

Nunes et al. [38] utilized the experimental data from Fernandes et al. [70] to estimate the web-crippling capacity of GFRP sections and demonstrated that progressive damage analysis provides more reliable predictions than the Tsai–Hill criterion. More recently, Asghar et al. [43] conducted a numerical study on the web-crippling of pultruded GFRP sections incorporating artificial intelligence. A finite element model was developed in ABAQUS CAE, validated against experimental data from rectangular hollow section specimens, and employed in an extensive parametric study. The findings revealed a direct correlation between web-crippling capacity and both section thickness and bearing length, whereas an inverse correlation was observed with section width, height, corner radius, and profile length. To refine predictive capabilities, gene expression programming (GEP)–based artificial intelligence was used to modify existing steel design guidelines, resulting in accurate predictions for GFRP RHS profiles. The proposed design rules were validated and shown to be reliable, representing a significant advancement in the field.

**Figure 25:**
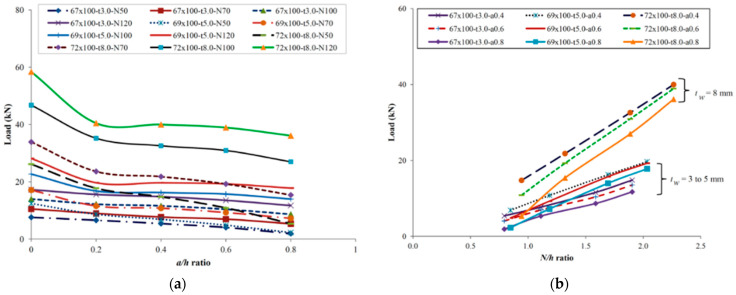
Variation of ultimate load with the parameters of Haloi et al. [50]: (**a**) the a/h parameter; (**b**) the N/h parameter.

Haloi et al. [66] reported their experimental studies and performed finite element modeling in ABAQUS. Both linear and nonlinear analyses were conducted, comparing ultimate loads, failure modes, and load–displacement curves. A total of 60 models were analyzed, varying key parameters such as perforation diameter (a/h) and support length (N/h) to evaluate their effects on web-crippling resistance. Finally, the authors proposed a resistance reduction factor to account for the influence of perforations. Rp = 0.81 − 0.40 + 0.02 ≤ 1, with the reduced ratio of a/h ≤ 0.8, N/h ≤ 2.14, and h/tw ≤ 20.33. The researchers thus validated the robustness of the proposed equation with a reliability index higher than the target (β = 3.71 ˃ 3.5). In another experimental study, Haloi et al. [50] reported that the a/h and N/h ratios were key parameters influencing the web-crippling behavior of GFRP sections (Figure 25a,b). For perforated profiles exposed to loads under ETF conditions, a strength reduction factor equation Rp = 0.66 − 0.59 + 0.13 ≤ 1 was proposed with a limit of a/h ≤ 0.8, h/tw ≤ 20.33, and N/h ≤ 2.14. It was validated with a reliability index (β = 3.54 ˃ 3.5).

**Figure 26:**
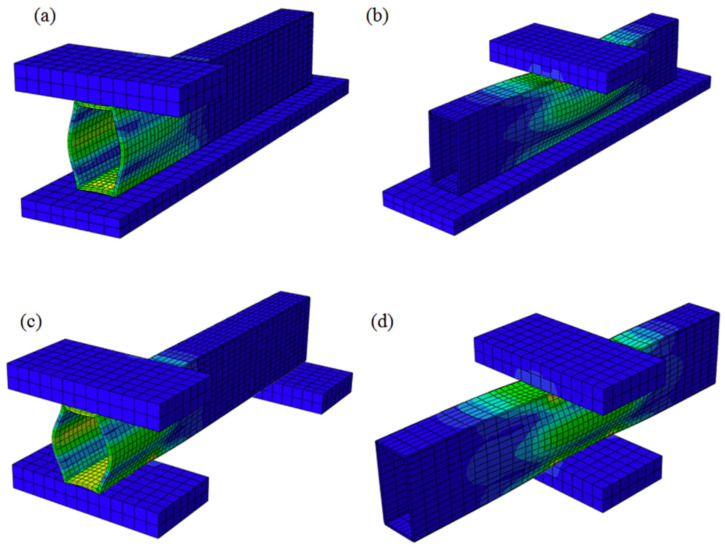
FE failures modes of the PGFRP rectangular hollow section under different loading conditions of Chen and Wang [54].

Chen and Wang [54] performed finite element modeling in ABAQUS, considering the meshes, element types, mechanical properties of materials (obtained from experimental tests), boundary conditions, and applied loads (Figure 26). The FE model was validated against experimental results, showing an average error of less than 3.35% in ultimate load predictions and accurately capturing the observed failure modes. The researchers proposed four distinct equations for estimating the ultimate capacities of hollow-section pultruded GFRP profiles under web-crippling for EG, ETF, IG, and ITF loading conditions.

Table 2 was derived using empirical coefficients and calibrated against experimental data in order to estimate the web crippling strength of rectangular hollow pultruded GFRP sections [54]. It was indicated that this formula provides a straightforward method to estimate the ultimate web strength with accuracy.

Haloi et al. [36] compared the web crippling strength prediction equations of Fernandes et al. [70], EC 3 ([73]) and NAS ([74]) for pultruded GFRP profiles, noting that Eurocode 3 and NAS equations are not suitable for anisotropic materials. Based on their findings, they developed six new equations (shown in Table 3) to estimate the web crippling capacity of pultruded GFRP I-sections under ETF and ITF loading conditions. According to their results of ETF loading, the ratio of test results-to-predicted value of modified EC3 [73], NAS [74] and Fernandes et al. [70] was on average 1.1, 1.25, and 1.21. These ratios are modified to 1.13, 1.13 and 1.08 for ITF loading.

Chen and Wang [53] conducted finite element modeling of the specimens in ABAQUS, incorporating material properties and mechanical characteristics, in order to compare the results with experimental tests. The study confirmed that the difference between experimental and FEA results was negligible. Equations for predicting the ultimate load of PGFRP I-sections are presented in Table 4. It was reported that the prediction to test capacity ratios were on average 0.89 with a standard deviation of 0.209.

Zhang and Chen [30] conducted finite element analyses on pultruded GFRP channel section specimens to simulate web-crippling behavior. The study validated the fracture modes and load–displacement curves, showing good agreement with experimental results, with an average error in ultimate loads of less than 3.35%. It was also observed that ultimate loads were higher under interior loading conditions (IG, ITF) than under end loading conditions (EG, ETF). Based on these findings, the authors proposed four equations to predict web-crippling behavior under varying loading conditions shown in Table 5. It was reported that the prediction to test capacity ratios were on average 0.92 with a standard deviation of 0.109.

## 4. Conclusions, Recommendations, and Future Work

The use of fiber-reinforced polymer (FRP) composites, particularly glass fiber-reinforced polymers (GFRP), has increased significantly in the construction sector due to their combined advantages of high strength, light weight, chemical resistance, and ease of installation. Pultruded GFRP sections, manufactured by embedding fiber reinforcements into resin matrices, exhibit excellent mechanical properties along the pultrusion direction. This limitation becomes particularly critical in applications involving high transverse loads or web openings. However, these profiles are susceptible to web crippling, especially under transverse loads, which limits their full mechanical potential.

Experimental investigations have identified multiple factors affecting web-crippling behavior, including profile geometry, bearing plate dimensions, loading type and position, web openings, and temperature effects. Short specimens, small bearing lengths, and perforations generally reduce ultimate load capacity and stiffness, whereas interior loading conditions produce higher resistance compared to end loading. Numerical and analytical studies, including finite element analyses and predictive models, have proven effective in capturing failure modes and estimating ultimate capacities. Nevertheless, reliance on steel-based design standards (eg., Eurocode 3, ASTM) introduces inaccuracies due to the orthotropic nature of GFRP.

Future design and assessment of pultruded GFRP profiles should consider their unique orthotropic behavior. Optimization of bearing plate dimensions, positions, and reinforcement of web openings with alternative materials can enhance structural performance. Additionally, careful selection of material orientation and web thickness may further improve resistance to web crippling. Advanced computational tools such as progressive damage modeling and AI-based parametric studies should complement experimental tests to provide reliable and efficient design guidance. Research should focus on multi-axial loading conditions, long-term performance under environmental and thermal cycles, and development of GFRP-specific design standards. Studies integrating experimental, numerical, and AI-based approaches under realistic service conditions will be particularly valuable. The development of GFRP-specific design guidelines is critical to avoid over- or underestimation of structural capacity. Long-term performance under environmental, chemical, and sustained loading conditions should be evaluated to ensure durability. Further studies on thermomechanical behavior, multi-scale modeling, and reinforcement strategies for web openings will support safer and more efficient applications of pultruded GFRP sections in structural engineering.

## Figures and Tables

**Table 1:** Summary of the experimental studies.

Reference and Authors	Loading Types	Profile Types	Observed Failures (As Described by Authors)
[37]	ETF, ITF, EG, IG	Square hollow pultruded GFRP sections	Initial web—flange junctions, (Web buckling and web crushing)
[15]	ETF, ITF	Pultruded GFRP channel section and built-up sections (I-section)	WFJ failure, Wedge-like cracking, Web cracking
[31]	ETF, ITF	GFRP Channel Sections	WFJ failure and WB failure
[53]	ETF, ITF, EG, IG	GFRP I-Sections	longitudinal bending cracks, bending wrinkling cracks and shear cracks.
[10]	ITF, ETF	GFRP I-Sections	Web crushing and Web buckling
[30]	ETF, ITF, EG, IG	GFRP Channel Sections	Bending main crack (on the web).
[54]	ETF, ITF, EG, IG	Rectangular hollow pultruded GFRP sections	WFJ failures, Bending crack (web bucking), longitudinal wrinkling cracks
[55]	Three-Point Bending	GFRP I-Sections	Wedge-like shear failure at the top web-flange junction, longitudinal cracking.
[56]	Three-Point Bending	GFRP I and W Section	Web-flange junction, Web bucking, Longitudinal cracking, web crushing.
[36,65]	ETF, ITF	GFRP Wide-Flange Sections	Material wrinkling, web–flange junction, Wedge shear failure, cracks longitudinal, web crushing.
[66]	ITF	GFRP Wide-Flange Sections	WJF failure, Wedge shear failure, longitudinal crack, shear crack.
[50]	ETF	GFRP Wide-Flange Sections	WJF failure, Wedge shear failure, longitudinal crack, shear crack, web crushing
[28]	ETF, ITF	GFRP Profiles with Various Fiber Layups	Web crushing and web buckling.
[49]	ETF, ITF, EG, IG	GFRP I-Sections with holes	Web–flange junction failure for circular holes; diagonal cracks for rectangular holes.
[11]	ETF, EG	GFRP I-Sections at elevated temperatures	Web–flange junction failure, longitudinal cracking at WFJ.
[67]	ETF, ITF	PGFRP Channel sections	Web—flange junction initial crack

**Table 2:** Equations for predicting ultimate load under ETF, ITF, EG, IG loading conditions of PGFRP rectangular hollow sections [54].

For EG loading condition	$N_{\mathrm{EG}}=0.8\left(6-\frac{H-2t_{f}}{130t_{w}}\right)\left(1+0.002\frac{{\left(b-100\right)}^{2}}{t_{w}}\right)t_{w}^{2}f_{y}$
For ETF loading condition	$N_{\mathrm{ETF}}=0.8\left(7-\frac{H-2t_{f}}{60t_{w}}\right)\left(1+0.002\frac{{\left(b-100\right)}^{2}}{t_{w}}\right)t_{w}^{2}f_{y}$
For IG loading condition	$N_{\mathrm{IG}}=0.8\left(15-\frac{H-2t_{f}}{50t_{w}}\right)\left(1+0.0002\frac{{\left(b-100\right)}^{2}}{t_{w}}\right)t_{w}^{2}f_{y}$
For ITF loading condition	$N_{\mathrm{ITF}}=0.8\left(16-\frac{H-2t_{f}}{16t_{w}}\right)\left(1+0.0002\frac{{\left(b-100\right)}^{2}}{t_{w}}\right)t_{w}^{2}f_{y}$

**Table 3:** Design formulas for estimating the web-crippling strength of PGFRP I-sections [36].

Based on:	P in ETF Loading Condition	P in ITF Loading Condition
EC 3	$0.252\left[12-\frac{h_{w}/t_{w}}{4.8}\right]\times \left[1+0.33\times \frac{N}{t_{w}}\right]t_{w}^{2}f_{cu,T}$	$0.31\left[17-\frac{h_{w}/t_{w}}{8}\right]\times \left[1+0.158\times \frac{N}{t_{w}}\right]t_{w}^{2}f_{cu,T}$
NAS	$0.164t_{w}^{2}f_{cu,T}\left(1-0.03\times \sqrt{\frac{r}{t_{w}}}\right)\times \left(1+36.5\times \sqrt{\frac{N}{t_{w}}}\right)\;\;\;\;\;\;\;\times \left(1-0.14\times \sqrt{\frac{h_{w}}{t_{w}}}\right)$	$1.23t_{w}^{2}f_{cu,T}\left(1-0.002\times \sqrt{\frac{r}{t_{w}}}\right)\times \left(1+4.0\times \sqrt{\frac{N}{t_{w}}}\right)\;\;\;\;\;\;\;\times \left(1-0.67\times \sqrt{\frac{h_{w}}{t_{w}}}\right)$
Fernandes et al. [70]	$0.0474{\;f}_{cu,T}h_{w}^{2}\left(1-0.58\times \sqrt{\frac{r}{t_{w}}}\right)\;\;\;\;\;\;\;\times \left(1+0.0036\times \frac{h_{w}^{2}N}{t_{w}^{3}}\right)\;\;\;\;\;\;\;\times \left(1-0.0634\times \frac{h_{w}}{t_{w}}\left(1-x_{F}\right)\right)$	$0.136{\;f}_{cu,T}h_{w}^{2}\left(1-0.5642\times \sqrt{\frac{r}{t_{w}}}\right)\;\;\;\;\;\;\;\times \left(1+0.0013\times \frac{h_{w}^{2}}{t_{w}^{3}}\right)\;\;\;\;\;\;\;\times \left(1-0.0606\times \frac{h_{w}}{t_{w}}\left(1-x_{F}\right)\right)$

**Table 4:** Equations for predicting ultimate load under ETF and ITF, EG and IG loading conditions for PGFRP I-sections [53].

For EG loading conditions	$\left(9-\frac{H-2t_{f}}{130t_{w}}\right)\left(1-0.01\frac{b}{t_{w}}\right)t_{w}^{2}f_{y}$
For IG loading conditions	$\left(9-\frac{H-2t_{f}}{50t_{w}}\right)\left(1-0.01\frac{b}{t_{w}}\right)t_{w}^{2}f_{y}$
For ETF loading conditions	$\left(9-\frac{H-2t_{f}}{60t_{w}}\right)\left(1-0.01\frac{b}{t_{w}}\right)t_{w}^{2}f_{y}$
For ITF loading conditions	$\left(12-\frac{(H-2t_{f})/t_{w}}{16}\right)\left(1-0.01\frac{b}{t_{w}}\right)t_{w}^{2}f_{y}$

**Table 5:** Equations for predicting ultimate load under ETF, ITF, EG and IG loading conditions for PGFRP channel sections [30].

For EG loading condition	$\left(1-\frac{\left(H-2t_{f}\right)/t_{w}}{130}\right)\left(1+0.004\times \frac{{\left(b-100\right)}^{2}}{t_{w}}\right)t_{w}^{2}f$
For ETF loading condition	$\left(3-\frac{(H-2t_{f})/t_{w}}{60}\right)\left(1+0.001\frac{b}{t_{w}}\right)t_{w}^{2}f$
For IG loading condition	$\left(6-\frac{(H-2t_{f})/t_{w}}{50}\right)\left(1-0.01\frac{b}{t_{w}}\right)\;t_{w}^{2}f$
For ITF loading condition	$\left(4-\frac{(H-2t_{f})/t_{w}}{16}\right)\left(1+0.001\frac{{\left(b-100\right)}^{2}}{t_{w}}\right)t_{w}^{2}f$

## Data Availability

No new data were created or analyzed in this study.
